# Evaluation of EUCAST Rapid Antimicrobial Susceptibility Testing for Gram-Negative ESKAPEEc Pathogens in Blood Cultures, with a Focus on Carbapenemase-Producing *Klebsiella pneumoniae* in a University Hospital in Palermo, Italy

**DOI:** 10.3390/antibiotics14121251

**Published:** 2025-12-11

**Authors:** Sara Cannella, Luca Pipitò, Martina Piazza, Domenico Graceffa, Rita Immordino, Roberta Virruso, Giovanni Maurizio Giammanco, Antonio Cascio, Celestino Bonura

**Affiliations:** 1 Microbiology and Virology Unit, AOU Policlinico “P. Giaccone”, 90133 Palermo, Italy; sara.cannella@policlinico.pa.it (S.C.); martina.piazza04@community.unipa.it (M.P.); domenico.graceffa@unipa.it (D.G.); rita.immordino@policlinico.pa.it (R.I.); roberta.virruso@policlinico.pa.it (R.V.); giovanni.giammanco@unipa.it (G.M.G.); 2 Department of Health Promotion, Mother and Child Care, Internal Medicine, and Medical Specialties “G D’Alessandro”, University of Palermo, 90133 Palermo, Italy; luca.pipito@community.unipa.it; 3 Infectious and Tropical Diseases Unit, Sicilian Regional Reference Center for the Fight Against AIDS, AOU Policlinico “P. Giaccone”, 90133 Palermo, Italy

**Keywords:** rapid antimicrobial susceptibility testing, EUCAST RAST, Gram-negative bacteria, bloodstream infections, *Klebsiella pneumoniae*, carbapenemases

## Abstract

**Background**: Rapid antimicrobial susceptibility testing (RAST) allows early detection of resistance directly from positive blood cultures, potentially improving outcomes in bloodstream infections (BSIs). We evaluated the performance of EUCAST RAST for Gram-negative ESKAPEEc pathogens and characterized carbapenemase genes in carbapenem-resistant *Klebsiella pneumoniae* (CRKP). **Methods**: A total of 354 positive blood cultures were screened, including 51 monomicrobial Gram-negative ESKAPEEc isolates. RAST results at 4, 6, 8, and 16–20 h were compared with standard antimicrobial susceptibility testing (AST) obtained using the BD Phoenix™ system. Categorical agreement (CA) and error frequency were calculated. Multiplex PCR and Sanger sequencing were performed on 15 CRKP isolates to identify carbapenemase genes and allelic variants. **Results**: 51 Gram-negative ESKAPEEc isolates met the inclusion criteria for RAST (15 *E. coli*, 19 *K. pneumoniae*, 11 *A. baumannii*, and 6 *P. aeruginosa*). Overall performance varied markedly by species and antibiotic. *E. coli* showed frequent unreadable or ATU zones at early timepoints and wide CA variability (50–100%), with high very major error (VME) rates for AMP, TZP, and CAZ, particularly at 6–8 h. *K. pneumoniae* displayed consistently high CA (mostly 100%) for carbapenems, CAZ, and TZP. *A. baumannii* demonstrated excellent agreement (100% for most agents), except for GEN at 6–8 h. *P. aeruginosa* could be evaluated only at 16–20 h, showing high CA for AMK, CAZ, and CIP; lower CA for MEM (83%); non-calculable CA for IMI due to universal ATU readings; and a CA value of 0% for TZP due to the predominance of the ATU results. VMEs ranged from 0% to 26.1% across species and reading times, but carbapenems did not generate VMEs. Molecular analysis revealed *bla_KPC_* in 66.7%, *bla_NDM_* in 46.7%, and *bla_OXA-48_* in 33.3% of isolates, with co-occurrence in several strains. Sequencing identified *bla_KPC-2_* and *bla_NDM-1_* as the predominant variants, with one isolate harboring *bla_NDM-5_*. **Conclusions**: EUCAST RAST markedly accelerates susceptibility reporting from positive blood cultures, but its accuracy is species- and time-dependent. Performance was excellent for *K. pneumoniae* (including CRKP) and *A. baumannii* and acceptable for *P. aeruginosa* at 16–20 h. In contrast, *E. coli* showed frequent ATU results at early timepoints and high ME/VME rates, making readings before 8 h unreliable for clinical decisions. Overall, RAST can effectively support rapid antimicrobial stewardship when species-specific limitations are recognized, and early-timepoint results are interpreted with caution.

## 1. Introduction

Timely administration of effective antimicrobial therapy is a critical determinant of outcomes in patients with bloodstream infections (BSIs) and sepsis [1]. However, conventional antimicrobial susceptibility testing (AST) typically requires 18–24 h or longer after colony isolation, delaying therapy optimization and potentially leading to prolonged use of broad-spectrum agents, suboptimal coverage, or overtreatment. To overcome these limitations, Rapid Antimicrobial Susceptibility Testing (RAST) was developed under the auspices of EUCAST (European Committee on Antimicrobial Susceptibility Testing) [2]. The EUCAST RAST method enables phenotypic antimicrobial susceptibility interpretation directly from positive blood culture bottles within a few hours, without the need for prior subculturing and plate isolation. In the RAST approach, a defined volume (100–150 µL) of broth from a positive blood culture is directly inoculated onto Mueller–Hinton agar without centrifugation or dilution; then antibiotic disks are applied. Plates are incubated under standard conditions and inspected at 4, 6, and 8 h using dedicated short-incubation breakpoints tailored to each time point [3]. Since April 2022, an extended reading window of 16–20 h has been introduced to accommodate laboratories with limited staffing, although the early readings remain the primary focus of the method [4].

During its validation, RAST demonstrated strong categorical agreement (CA) with standard disk diffusion methods [5]. Beyond analytical performance, increasing evidence supports its clinical utility: RAST can shorten the time to susceptibility reporting by many hours (often by more than 24 h), enabling earlier de-escalation or escalation of therapy, especially in settings with a high prevalence of antimicrobial resistance. The ESKAPEEc pathogens (*Enterococcus faecium*, *Staphylococcus aureus*, *Klebsiella pneumoniae*, *Acinetobacter baumannii*, *Pseudomonas aeruginosa*, *Enterobacter* spp., and *Escherichia coli*) are major causes of healthcare-associated infections and are well known for their ability to “escape” multiple classes of antibiotics [6]. Due to their high levels of antimicrobial resistance and clinical relevance, the World Health Organization has classified them as high-priority targets of novel antimicrobial strategies [7]. In this context, the EUCAST RAST method represents a valuable diagnostic tool for the early detection of resistance directly from positive blood cultures, supporting timely optimization of antimicrobial therapy [8]. When combined with interpretive reading (e.g., double-disk synergy test), RAST screening can detect extended-spectrum β-lactamases (ESBLs) or carbapenemase producers in Enterobacterales as early as 4–8 h, offering early guidance on resistance phenotypes [8]. Furthermore, a fully automated EUCAST RAST can substantially improve laboratory workflow by reducing hands-on time and eliminating the constraints of manual time-point reading [9].

In this study, we report the experience of the Microbiology Laboratory at the University Hospital “Paolo Giaccone” in Palermo, Italy, evaluating the concordance between EUCAST RAST and the BD Phoenix automated system for positive blood cultures in which MALDI-TOF MS identified Gram-negative ESKAPEEc pathogens. In addition, we characterized carbapenemase genes in carbapenem-resistant *Klebsiella pneumoniae* (CRKP).

## 2. Results

### 2.1. Rapid Antimicrobial Susceptibility Testing and Categorical Agreement

A total of 354 positive blood culture specimens from patients with suspected BSIs were evaluated. Following Gram staining, 51 specimens (14.4%) producing a single Gram-negative ESKAPEEc identification met the inclusion criteria and were considered for RAST. The remaining 303 samples were excluded according to predefined criteria specified in the Section 4. The 51 interpretable cultures included 15 *E. coli* strains, 19 *K. pneumoniae* strains, 11 *A. baumannii* strains, and 6 *P. aeruginosa* strains. The data for each isolate reading at each time point, the CA rates, and the discrepancies (including associated error types) are summarized in Table 1, Table 2, Table 3 and Table 4.

For *E. coli*, 11 isolates were evaluated at 4, 6, and 8 h, while 4 isolates were evaluated only at 16/20 h. For MEM and IMI, a CA of 100% was observed between RAST and the reference method across all reading times, except for MEM at 16/20 h, where CA decreased to 75%. However, in most cases, the inhibition zone for carbapenem was not readable at 4 h. Among the other antibiotics, AMK showed 100% CA at 4, 8, and 16/20 h, and 88% at 6 h. AMP exhibited 75% CA at 4 and 6 h, 82% at 8 h, and 100% 16/20 h. For TZP, the CA values were 100%, 88%, 91%, and 100% at 4, 6, 8, and 16/20 h, respectively. CAZ demonstrated 100%, 88%, 91%, and 75% CA at the same time points. CIP and GEN showed CAs of 100%, 88%, 91%, and 100% at 4, 6, 8, and 16/20 h, respectively. TMP-SMX presented the lowest values, with CA of 50%, 50%, 55%, and 75% at 4, 6, 8, and 16/20 h, respectively. For most antibiotics inhibition zones were frequently unreadable at 4–6 h.

For *K. pneumoniae*, 10 isolates were evaluated at 4, 6, and 8 h, while 9 isolates were evaluated at 16/20 h only. CA was 100% for IMI, MEM, CAZ, and TZP across all incubation periods. Other antibiotics showed slightly lower agreement. For CIP, CA was 100% at 4 h, 90% at 6 and 8 h, and 89% for the nine isolates read at 16/20 h. GEN and AMK displayed 100% CA at 4 h, 90% at 6 and 8 h, and 89% for AMK and 100% for GEN at 16/20 h. TMP-SMX showed the lowest agreement rates, with 90% at 4 h, 6 and 8 h, and 67% at 16/20 h.

For *A. baumannii*, 6 out of 11 isolates were evaluated at 4, 6, and 8 h, while 5 isolates were evaluated at 16/20 h. Complete CA (100%) was observed at all reading times for IMI, MEM, CIP, AMK, and TMP-SMX. Some discrepancies were detected for GEN, which showed 100% CA at 4 and 16/20 h, and 83% at 6 and 8 h.

For *P. aeruginosa*, all isolates were evaluated at 16–20 h, and CA (100%) was observed with AMK, CAZ, and CIP. For MEM the CA values was 83%. ATU readings occurred in all *P. aeruginosa* isolates for IMI, making the CA not calculable. For TZP, 4 out of 6 readings fell within the ATU, and the CA was 0%. In Table 5 CA ranges for main pathogen-antibiotic combinations are summarized.

VME, ME, and mE frequencies for each reading time are presented in Table 1, Table 2, Table 3 and Table 4. VME rates ranged from 0.0% to 26.1%, depending on the specific microorganism and the reading time. However, in all cases, VMEs did not involve antibiotics typically used for the treatment of bacteremia, except for AMP, TZP, and CAZ for *E. coli*, for which high VME rates were observed. Carbapenems did not yield any error type, except in two instances (one mE for *E. coli* and one ME for *P. aeruginosa* at 16–20 h). Rates of VME and ME according to reading times and microorganisms are reported in Table 6.

### 2.2. Carbapenem-Resistant K. pneumoniae and Carbapenemase-Encoding Genes Characterization

The 15 CRKP detected by RAST and phoenix system were analyzed to detect the presence of carbapenemase-encoding genes. PCR amplification followed by gel electrophoresis revealed that 10 out of 15 isolates (66.7%) were positive for the *bla_KPC_* gene, 7/15 (46.7%) for *bla_NDM_*, and 5/15 (33.3%) for *bla_OXA_*_-48_. The *bla_IMP_* and *bla_VIM_* genes were not detected in any isolate. Co-occurrence of carbapenemase genes was observed in several strains: *bla_OXA-48_* and *bla_NDM_* were detected together in four isolates, *bla_KPC_* and *bla_NDM_* in two, and *bla_KPC_* and *bla_OXA-48_* in one isolate. Sequencing of *bla_KPC_* and *bla_NDM_* genes was performed to determine the allelic variants. BLAST analysis revealed that all *bla_KPC_*-positive isolates carried the *bla_KPC-2_* carbapenemase variant, showing sequence similarity at the nucleotide level ranging from 81% to 98% compared with reference sequences available in the NCBI database. Similarly, all *bla_NDM_*-positive isolates were identified as the *bla_NDM-1_* variant, with similarity values to reference sequences between 81% and 95%, except for one isolate that harbored the *bla_NDM-5_* variant (96% identity to the reference sequence).

## 3. Discussion

In this study, the EUCAST RAST was evaluated on 51 monomicrobial Gram-negative ESKAPEEc isolates obtained from positive blood cultures. Our results demonstrate a high level of CA between RAST and the reference BD Phoenix system across most species–antibiotic combinations. These findings confirm that reliable susceptibility results can be obtained within 4–8 h of culture positivity for the majority of clinically relevant Gram-negative isolates, and at 16–20 h for *P. aeruginosa*.

Notably, for *E. coli*, early readings often fell into the ATU. This substantially reduced the proportion of actionable early results for this species. Nevertheless, when *E. coli* readings were interpretable, RAST demonstrated high CA for carbapenems, TZP, CAZ, and aminoglycosides, while TMP-SMX consistently showed lower agreement.

For *K. pneumoniae*, including CRKP isolates, RAST performed robustly at early timepoints with excellent CA and no VME, supporting the strong suitability of RAST for accelerated detection of carbapenem resistance in this species.

For *A. baumannii*, the method demonstrated consistent accuracy, with 100% CA for most antibiotics tested and only minor discrepancies for GEN. In *P. aeruginosa*, complete CA was obtained for AMK, CAZ, and CIP, while slightly lower values were observed for MEM. For IMI, the CA could not be calculated because all readings fell within the ATU. For TZP, the CA was 0% because 4 out of 6 readings were in the ATU, and the 2 results classified as R by the RAST were interpreted as I by the Phoenix system.

In partial contrast with our findings, based on blood cultures collected from 40 laboratories in northern Europe and 15 in southern Europe, Åkerlund et al. first demonstrated that EUCAST RAST provides accurate results for *E. coli* and *K. pneumoniae* within 4–8 h, with CA rates above 90% for β-lactams and aminoglycosides, while reliable results for *P. aeruginosa* were achieved only after 6–8 h of incubation [5]. Subsequent studies confirmed these findings, showing high concordance between RAST and standard AST and highlighting that early RAST results can guide targeted antimicrobial therapy, allowing either de-escalation or escalation significantly earlier than conventional AST in BSIs [10]. In a case–control study, Cardot et al. reported that in the RAST group, effective antibiotic therapy was prescribed in 100% of patients on the day of blood culture positivity [11].

CA of the RAST method with standard AST exceeded 90% at 6 h for most antibiotics in the study by Özgen et al., which included BSIs caused by *E. coli*, *K. pneumoniae*, *P. aeruginosa*, and *A. baumannii*. Lower CA and higher ATU rates were observed for TZP at 6 h, particularly for *E. coli*, with six major errors reported [12]. Bianco et al. found that a high number of readings in the ATU category were produced for TZP in *E. coli* at 4 h, 6 h, and 8 h of incubation, which significantly decreased at 16–20 h. ATU rates exceeding 10% for TZP were also reported for *K. pneumoniae* and *P. aeruginosa* [13]. Conversely, Tian et al. found 100% CA for TZP in *E. coli* at all time points, with major errors limited to TMP-SMX, while *K. pneumoniae* showed complete concordance with standard AST [14]. For *P. aeruginosa*, Lebreton et al. reported significant rates of VME and ME after 6 h, especially for CAZ, TZP, and MEM, which increased further with an extended incubation to 8 h [15].

In carbapenem-resistant *K. pneumoniae* isolates, Xu et al. observed low CA for MEM and high MEs at the 6 h reading, which improved when the incubation was extended to 8 h, but with a high proportion of strains within the ATU [16].

These results collectively demonstrate the high reliability of the RAST method while also highlighting inter-laboratory variability despite EUCAST standardization.

In our study, the performance for *E. coli* was noticeably less robust than in several of these reports. Although high CA values were observed for some agents when inhibition zones were interpretable, early readings (4–6 h) were frequently ATU or unreadable, and VME rates reached up to 26%. These findings indicate that early RAST readings for *E. coli* in our setting cannot be considered reliably actionable and require cautious interpretation. For *P. aeruginosa*, results were strongly affected by the high frequency of ATU readings, and overall interpretation was limited by the small number of isolates (*n* = 6).

Data on carbapenem-resistant *K. pneumoniae* in our study were more favorable. In all CRKP isolates, resistance was accurately identified by RAST within 8 h, and in nearly all cases within 4 h, without discrepancies. Furthermore, the absence of false-susceptible results for carbapenems is clinically reassuring, given the high prevalence of carbapenem-resistant Enterobacterales in our hospital and the associated increased mortality in sepsis [17,18,19]. These findings highlight the potential of RAST to enable rapid detection and prompt adjustment of antimicrobial therapy. EUCAST RAST showed excellent performance in determining ceftazidime-avibactam (CAZ-AVI) susceptibility, achieving 100% CA and no errors at each time point, making it an effective tool for rapid management of carbapenem-resistant *K. pneumoniae* BSIs [16]. In our study, however, CAZ-AVI susceptibility was not assessed by RAST. We performed molecular characterization of the carbapenem-resistant *K. pneumoniae* isolates, revealing a predominance of the *bla_KPC-2_* and *bla_NDM-1_* carbapenemase variants, in line with current epidemiological trends observed in several Italian healthcare settings [20]. The co-occurrence of multiple carbapenemase genes in some isolates (e.g., *bla_NDM_* and *bla_OXA_*_-48_ or *bla_KPC_* and *bla_NDM_*) further highlights the genetic complexity and potential for horizontal gene transfer among circulating CRKP strains [21]. From a clinical perspective, the concordance between RAST results and the genotypic detection of carbapenemase genes supports the reliability of RAST in rapidly identifying carbapenem resistance [16]. Early phenotypic detection (in most cases within 4 h) may therefore provide timely guidance for escalating therapy and confirm molecular detection of carbapenemase genes. The predominance of KPC-2-producing isolates also aligns with the observed phenotypic susceptibility patterns, confirming the strong correlation between RAST results and underlying resistance mechanisms.

### Strengths and Limitations

Finally, our data reinforce the practical feasibility of implementing RAST in routine microbiology workflows. The use of validated systems such as BD BACTEC FX and MALDI-TOF MS for rapid identification, combined with EUCAST-compliant direct inoculation procedures, allowed our laboratory to produce preliminary AST results within a single working shift. The inclusion of a detailed molecular characterization of carbapenemase-producing *K. pneumoniae*, including allelic variant identification, further strengthens the study by linking phenotypic performance with underlying resistance mechanisms. Moreover, the analysis covers multiple Gram-negative ESKAPEEc species, thereby improving its applicability in routine clinical microbiology.

However, the study has several limitations that should be acknowledged. Its single-center design and limited sample size, particularly when stratified by species and early time-point readings, may reduce the generalizability of the findings to laboratories with different epidemiological contexts, workflows, or staffing models. RAST readings could not always be performed at all recommended time points due to limited laboratory operating hours. Similar logistical constraints have been reported in other studies [22]. Nonetheless, the flexibility of EUCAST RAST, which allows extended incubation of 16–20 h, can mitigate this limitation and increase applicability in laboratories with reduced operating shifts [4]. In addition, clinical breakpoints for some antibiotic–species combinations resulted in high ATU or unreadable zone frequencies at early readings, particularly for *E. coli* and *P. aeruginosa*, limiting interpretability. Finally, the study did not include evaluation of certain important therapeutic agents, such as ceftazidime–avibactam, preventing assessment of RAST performance for these clinically significant drug options.

## 4. Materials and Methods

### 4.1. Rapid Antimicrobial Susceptibility Testing

This single-center, prospective descriptive study was conducted between 5 October 2024, and 31 July 2025, at the University Hospital “Paolo Giaccone” in Palermo, Italy. Two to three sets of blood cultures were collected from patients aged ≥18 years with suspected bloodstream infections.

**Figure 1:**
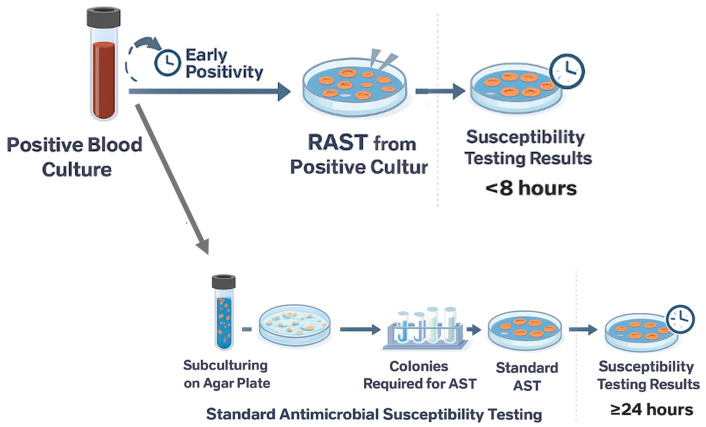
Workflow comparison between EUCAST Rapid Antimicrobial Susceptibility Testing (RAST) and standard AST directly from positive blood cultures. RAST is performed immediately from the positive blood culture bottle, allowing interpretation of inhibition zones within 4–8 h and enabling early reporting of susceptibility results. In contrast, standard AST requires subculturing and overnight incubation to obtain isolated colonies, delaying susceptibility results to ≥24 h. The schematic highlights how RAST shortens the diagnostic timeline by bypassing the colony-isolation step.

Blood specimens were processed using the BD BACTEC FX automated blood culture system (Becton Dickinson, Franklin Lakes, NJ, USA). Blood culture bottles were incubated for up to 5 days to detect microbial growth. Upon positivity, microbial growth was rapidly identified using MALDI-TOF MS (VITEK MS, bioMérieux, Marcy-l’Étoile, France, Matrix-Assisted Laser Desorption/Ionization–Time of Flight Mass Spectrometry). When MALDI-TOF MS presumptive identification indicated a single Gram-negative ESKAPEEc pathogen, both EUCAST RAST and reference broth microdilution antimicrobial susceptibility testing using the BD Phoenix system were performed in parallel from the positive blood culture, Figure 1. Blood cultures yielding polymicrobial growth, fungi, and nonESKAPEEc organisms were excluded.

RAST was carried out according to the EUCAST RAST guidelines (Version 6.1) [3]. A volume of 100–150 µL was taken directly from the positive blood culture bottle (validated systems: BD, bioMérieux, and Thermo Fisher, Waltham, MA, USA) within 0–18 h of the positive signal and inoculated directly onto Mueller–Hinton agar plates for disk diffusion testing, without centrifugation or dilution of the inoculum. Plates were streaked according to the EUCAST disk diffusion procedure, and the following antibiotics disks were applied depending on the isolate: ceftazidime (CAZ), piperacillin/tazobactam (TZP), meropenem (MEM), imipenem (IMI), ciprofloxacin (CIP), amikacin (AMK), gentamicin (GEN), trimethoprim-sulfamethoxazole (TMP-SMX), colistin (CST), ampicillin (AMP). Plates were incubated at 35 ± 1 °C and read at 4, 6, and 8 h.

Plates not readable at these time points because of laboratory operating hours were read at 16–20 h. For *P. aeruginosa*, only the 16–20 h reading was considered. After each reading, plates were re-incubated within 10 min. Zone diameters were measured from the front of the plate, after removing the lid, only when a clear and distinct inhibition edge was visible. Plates lacking well-defined zone edges were re-incubated until the next scheduled reading. Results were interpreted using RAST-specific breakpoints defined for each bacterial species and incubation time, as provided in the dedicated EUCAST RAST breakpoint tables. RAST results were classified as: inhibition zone not readable (poor growth), ATU (area of technical uncertainty, where no categorization can be offered), S (susceptible), or R (resistant) at each incubation interval.

Reference AST was performed using the BD Phoenix™ Automated Microbiology System (Becton Dickinson, Franklin Lakes, NJ, USA). Identification was based on biochemical profiles, and antimicrobial susceptibility testing relied on analysis of bacterial growth kinetics in the presence of predefined antibiotic concentrations. Results are interpreted automatically according to EUCAST clinical breakpoints, providing minimum inhibitory concentration (MIC) values and categorical interpretations (S, increased exposure [I], R). Agreement between the EUCAST RAST and Phoenix results was assessed for each bacterial species. The comparisons of RAST results with conventional incubation AST were categorized as follows: categorical agreement (CA), very major error (VME, indicating false S), major error (ME, indicating false R), and minor error (mE, indicating I isolates misclassified as S or R).

VME rates were calculated by dividing the number of S in RAST and R in Phoenix by the total R number in Phoenix. ME rates were calculated by dividing the number of R in RAST and S in Phoenix by the total S number in Phoenix.

Data were summarized as absolute numbers and relative frequencies (%). CA was calculated as = (Number of categorical result matches/Total tested) × 100. Since the I category does not apply to RAST, EUCAST introduced the concept of ATU, and unreadable inhibition zones were excluded from CA calculations.

### 4.2. Multiplex PCR Technique

The main carbapenemase-encoding genes (*bla_KPC_*, *bla_NDM_*, *bla_VIM_*, *bla_IMP_*, *bla_OXA-48_*) were investigated in CRKP. Detection was performed using two multiplex polymerase chain reaction (PCR) assays, as described by Poirel et al. (2011) [23]. PCRs were carried out in a final volume of 50 μL. The first multiplex mix targeted *blaIMP* and *blaVIM* genes, while the second amplified *bla_KPC_*, *bla_NDM_*, and *bla_OXA_*_-48_. Reaction mixtures contained molecular-grade water, buffer, MgCl_2_ (25 mM), dNTPs, specific primers (forward and reverse), Taq DNA polymerase, and 5 μL of DNA template. PCR cycling conditions were as follows: initial denaturation at 94 °C for 10 min; 36 cycles of 94 °C for 30 s, 52 °C for 40 s, and 72 °C for 50 s; and a final extension at 72 °C for 5 min. PCR products were visualized by 2% agarose gel electrophoresis in 1× TAE buffer containing SYBR^®^ Safe DNA gel stain and examined under UV light. The expected amplicon sizes were: *bla_KPC_* (798 bp), *bla_NDM_* (621 bp), *blaIMP* (232 bp), *blaVIM* (390 bp), and *bla_OXA-48_* (438 bp). All PCR-positive amplicons underwent Sanger sequencing. PCR products were purified and concentrated using Amicon spin columns, then used as templates for cycle sequencing reactions with the ABI BigDye Terminator v1.1 Kit in a final volume of 20 μL (14 μL of H_2_O, 3 μL of BigDye, 1 μL of the forward primer at 5 μM, and 2 μL of the purified template). Sequencing products were further purified using CentriSep columns and analyzed by capillary electrophoresis on an ABI Prism 310 Genetic Analyzer. Sequence chromatograms were processed with Sequencing Analysis and BioEdit Sequence Alignment Editor software v7.7, and the resulting FASTA files were compared to reference sequences in the NCBI database using BLAST (Basic Local Alignment Search Tool) to confirm gene identity [24].

## 5. Conclusions

EUCAST RAST shortens the time to susceptibility reporting directly from positive blood cultures, but its performance is highly species- and time-dependent. A critical analysis of discrepancy rates shows that, while some pathogens exhibit excellent performance, other pathogens, most notably *E. coli*, display relevant limitations that must be considered in routine clinical practice.

For *E. coli*, early readings (4–6 h) were frequently ATU due to poorly defined inhibition zones, and among interpretable results, the rates of ME and VME were substantially higher than desirable, reaching values of up to 26% for specific drug–timepoint combinations. RAST for *E. coli* cannot be considered reliably actionable at early timepoints, and 8 h readings should be viewed as the minimum acceptable threshold for clinical decision-making. Even at 8 h, caution is warranted for agents showing low CA or persistent discrepancies.

For *K. pneumoniae*, including CRKP, RAST demonstrated excellent CA across most agents and timepoints, correctly identifying carbapenem resistance without VME. Thus, RAST can be confidently recommended for routine rapid reporting for *K. pneumoniae*, particularly for early recognition of carbapenem resistance.

For *A. baumannii*, RAST showed high CA for most agents, with isolated VME for GEN. For *P. aeruginosa*, readings were performed only at 16–20 h, displaying elevated CA and no VME detection.

In conclusion, EUCAST RAST offers substantial advantages in accelerating AST, but its reliability varies significantly by species and antibiotic. Incorporating RAST into the diagnostic workflow can reduce turnaround time and support timely optimization of antimicrobial therapy, contributing to improved antimicrobial stewardship in acute-care settings.

## Figures and Tables

**Table 1:** RAST and BD Phoenix™ Automated Microbiology System results for *E. coli*.

	AMK				AMP				TZP				CAZ				IMI				MEM				CIP				GEN				TMP-SMX			
	4 h	6 h	8 h	16/20 h	4 h	6 h	8 h	16/20 h	4 h	6 h	8 h	16/20 h	4 h	6 h	8 h	16/20 h	4 h	6 h	8 h	16/20 h	4 h	6 h	8 h	16/20 h	4 h	6 h	8 h	16/20 h	4 h	6 h	8 h	16/20 h	4 h	6 h	8 h	16/20 h
N. isolates 4/6/8 h = 11																																				
Resistant	0	1	0	/	3	5	6	/	0	0	0	/	0	2	2	/	0	0	0	/	0	0	0	/	0	2	2	/	0	0	0	/	1	1	2	/
ATU	0	0	0	/	0	0	0	/	1	2	2	/	0	0	0	/	0	0	0	/	0	0	0	/	0	0	0	/	0	0	0	/	0	0	0	/
Susceptible	3	7	11	/	1	3	5	/	2	6	9	/	3	6	9	/	3	8	11	/	4	8	11	/	4	6	9	/	4	8	11	/	3	7	9	/
Inhibition zone not readable	8	3	0	/	7	3	0	/	8	3	0	/	8	3	0	/	8	3	0	/	7	3	0	/	7	3	0	/	7	3	0	/	7	3	0	/
N. isolates 16/20 h = 4																																				
Resistant	/	/	/	0	/	/	/	2	/	/	/	0	/	/	/	0	/	/	/	0	/	/	/	0	/	/	/	1	/	/	/	0	/	/	/	3
ATU	/	/	/	0	/	/	/	1	/	/	/	0	/	/	/	0	/	/	/	0	/	/	/	0	/	/	/	0	/	/	/	0	/	/	/	0
Susceptible	/	/	/	4	/	/	/	1	/	/	/	4	/	/	/	4	/	/	/	4	/	/	/	4	/	/	/	3	/	/	/	4	/	/	/	1
Inhibition zone not readable	/	/	/	0	/	/	/	0	/	/	/	0	/	/	/	0	/	/	/	0	/	/	/	0	/	/	/	0	/	/	/	0	/	/	/	0
Phoenix																																				
Resistant	/	/	/	0	/	/	/	10	/	/	/	1	/	/	/	3	/	/	/	0	/	/	/	0	/	/	/	3	/	/	/	1	/	/	/	5
Increased exposure	/	/	/	0	/	/	/	0	/	/	/	0	/	/	/	1	/	/	/	0	/	/	/	1	/	/	/	1	/	/	/	0	/	/	/	2
Susceptible	/	/	/	15	/	/	/	5	/	/	/	14	/	/	/	11	/	/	/	15	/	/	/	14	/	/	/	11	/	/	/	14	/	/	/	7
Categorical agreement (%) ^a^	100	88	100	100	75	75	82	100	100	88	91	100	100	88	91	75	100	100	100	100	100	100	100	75	100	88	91	100	100	88	91	100	50	50	55	75
Minor error	0	0	0	0	0	0	0	0	0	0	0	0	0	1	1	0	0	0	0	0	0	0	0	1	0	1	1	0	0	0	0	0	1	2	2	0
Major error	0	1	0	0	0	0	0	0	0	0	0	0	0	0	0	0	0	0	0	0	0	0	0	0	0	0	0	0	0	0	0	0	0	0	1	1
Very major error	0	0	0	0	1	2	2	0	0	1	1	0	0	0	0	1	0	0	0	0	0	0	0	0	0	0	0	0	0	1	1	0	1	2	2	0

^a^ Agreement between RAST and the reference method (Phoenix). Ceftazidime (CAZ), piperacillin/tazobactam (TZP), meropenem (MEM), imipenem (IMI), ciprofloxacin (CIP), amikacin (AMK), gentamicin (GEN), trimethoprim-sulfamethoxazole (TMP-SMX), ampicillin (AMP), area of technical uncertainty (ATU).

**Table 2:** RAST and BD Phoenix™ Automated Microbiology System results for *K. pneumoniae*.

	TZP				CAZ				IMI				MEM				CIP				GEN				AMK				TMP-SMX			
	4 h	6 h	8 h	16/20 h	4 h	6 h	8 h	16/20 h	4 h	6 h	8 h	16/20 h	4 h	6 h	8 h	16/20 h	4 h	6 h	8 h	16/20 h	4 h	6 h	8 h	16/20 h	4 h	6 h	8 h	16/20 h	4 h	6 h	8 h	16/20 h
N. isolates 4/6/8 h = 10																																
Resistant	7	9	9	/	7	9	9	/	7	8	8	/	7	8	8	/	7	8	8	/	4	4	4	/	5	5	5	/	5	6	6	/
ATU	0	0	0	/	1	1	1	/	0	0	0	/	0	0	0	/	1	1	1	/	0	0	0	/	0	0	0	/	0	0	0	/
Susceptible	1	1	1	/	0	0	0	/	1	2	2	/	1	2	2	/	0	1	1	/	4	6	6	/	3	5	5	/	0	4	4	/
Inhibition zone not readable	2	0	0	/	2	0	0	/	2	0	0	/	2	0	0	/	2	0	0	/	2	0	0	/	2	0	0	/	5	0	0	/
N. isolates 16/20 h = 9																																
Resistant	/	/	/	8	/	/	/	7	/	/	/	7	/	/	/	7	/	/	/	9	/	/	/	4	/	/	/	4	/	/	/	7
ATU	/	/	/	0	/	/	/	1	/	/	/	0	/	/	/	0	/	/	/	0	/	/	/	1	/	/	/	0	/	/	/	0
Susceptible	/	/	/	1	/	/	/	1	/	/	/	2	/	/	/	2	/	/	/	0	/	/	/	4	/	/	/	5	/	/	/	2
Inhibition zone not readable	/	/	/	0	/	/	/	0	/	/	/	0	/	/	/	0	/	/	/	0	/	/	/	0	/	/	/	0	/	/	/	
Phoenix																																
Resistant	/	/	/	17	/	/	/	17	/	/	/	15	/	/	/	15	/	/	/	16	/	/	/	9	/	/	/	11	/	/	/	9
Increased exposure	/	/	/	0	/	/	/	1	/	/	/	0	/	/	/	0	/	/	/	1	/	/	/	0	/	/	/	0	/	/	/	0
Susceptible	/	/	/	2	/	/	/	1	/	/	/	4	/	/	/	4	/	/	/	2	/	/	/	10	/	/	/	8	/	/	/	10
Categorical agreement (%) ^a^	100	100	100	100	100	100	100	100	100	100	100	100	100	100	100	100	100	90	90	89	100	90	90	100	100	90	90	89	90	90	90	67
Minor error	0	0	0	0	0	0	0	0	0	0	0	0	0	0	0	0	0	1	1	0	0	0	0	0	0	0	0	0	0	0	0	0
Major error	0	0	0	0	0	0	0	0	0	0	0	0	0	0	0	0	0	0	0	1	0	0	0	0	0	0	0	0	1	1	1	3
Very major error	0	0	0	0	0	0	0	0	0	0	0	0	0	0	0	0	0	0	0	0	0	1	1	0	0	1	1	1	0	0	0	0

^a^ Agreement between RAST and the reference method (Phoenix). Ceftazidime (CAZ), piperacillin/tazobactam (TZP), meropenem (MEM), imipenem (IMI), ciprofloxacin (CIP), amikacin (AMK), gentamicin (GEN), trimethoprim-sulfamethoxazole (TMP-SMX), area of technical uncertainty (ATU).

**Table 3:** RAST and BD Phoenix™ Automated Microbiology System results for *A. baumannii*.

	IMI				MEM				CIP				AMK				GEN				TMP-SMX			
	4 h	6 h	8 h	16/20 h	4 h	6 h	8 h	16/20 h	4 h	6 h	8 h	16/20 h	4 h	6 h	8 h	16/20 h	4 h	6 h	8 h	16/20 h	4 h	6 h	8 h	16/20 h
N. isolates 4/6/8 h = 6																								
Resistant	4	6	6	/	4	6	6	/	4	6	6	/	4	6	6	/	4	5	5	/	4	6	6	/
ATU	0	0	0	/	0	0	0	/	0	0	0	/	0	0	0	/	0	0	0	/	0	0	0	/
Susceptible	0	0	0	/	0	0	0	/	0	0	0	/	0	0	0	/	0	1	1	/	0	0	0	/
Inhibition zone not readable	2	0	0	/	2	0	0	/	2	0	0	/	2	0	0	/	2	0	0	/	2	0	0	/
N. isolates 16/20 h = 5																								
Resistant	/	/	/	5	/	/	/	5	/	/	/	5	/	/	/	5	/	/	/	5	/	/	/	5
ATU	/	/	/	0	/	/	/	0	/	/	/	0	/	/	/	0	/	/	/	0	/	/	/	0
Susceptible	/	/	/	0	/	/	/	0	/	/	/	0	/	/	/	0	/	/	/	0	/	/	/	0
Inhibition zone not readable	/	/	/	0	/	/	/	0	/	/	/	0	/	/	/	0	/	/	/	0	/	/	/	0
Phoenix																								
Resistant	/	/	/	11	/	/	/	11	/	/	/	11	/	/	/	11	/	/	/	11	/	/	/	11
Increased exposure	/	/	/	0	/	/	/	0	/	/	/	0	/	/	/	0	/	/	/	0	/	/	/	0
Susceptible	/	/	/	0	/	/	/	0	/	/	/	0	/	/	/	0	/	/	/	0	/	/	/	0
Categorical agreement (%) ^a^	100	100	100	100	100	100	100	100	100	100	100	100	100	100	100	100	100	83	83	100	100	100	100	100
Minor error	0	0	0	0	0	0	0	0	0	0	0	0	0	0	0	0	0	0	0	0	0	0	0	0
Major error	0	0	0	0	0	0	0	0	0	0	0	0	0	0	0	0	0	0	0	0	0	0	0	0
Very major error	0	0	0	0	0	0	0	0	0	0	0	0	0	0	0	0	0	1	1	0	0	0	0	0

^a^ Agreement between RAST and the reference method (Phoenix). Meropenem (MEM), imipenem (IMI), ciprofloxacin (CIP), amikacin (AMK), gentamicin (GEN), trimethoprim-sulfamethoxazole (TMP-SMX), colistin (CST), area of technical uncertainty (ATU).

**Table 4:** RAST and BD Phoenix™ Automated Microbiology System results for *P. aeruginosa*.

	AMK				IMI				MEM				CAZ				TZP				CIP			
	4 h	6 h	8 h	16/20 h	4 h	6 h	8 h	16/20 h	4 h	6 h	8 h	16/20 h	4 h	6 h	8 h	16/20 h	4 h	6 h	8 h	16/20 h	4 h	6 h	8 h	16/20 h
N. isolates 16/20 h = 6																								
Resistant	/	0	0	0	/	0	0	0	/	0	0	1	/	0	0	3	/	0	0	2	/	0	0	1
ATU	/	0	0	0	/	0	0	6	/	0	0	0	/	0	0	3	/	0	0	4	/	0	0	5
Susceptible	/	0	0	6	/	0	0	0	/	0	0	5	/	0	0	0	/	0	0	0	/	0	0	0
Inhibition zone not readable	/	0	0	0	/	0	0	0	/	0	0	0	/	0	0	0	/	0	0	0	/	0	0	0
Phoenix																								
Resistant	/	/	/	0	/	/	/	0	/	/	/	0	/	/	/	3	/	/	/	0	/	/	/	1
Increased exposure	/	/	/	0	/	/	/	6	/	/	/	0	/	/	/	3	/	/	/	6	/	/	/	5
Susceptible	/	/	/	6	/	/	/	0	/	/	/	6	/	/	/	0	/	/	/	0	/	/	/	0
Categorical agreement (%) ^a^	/	/	/	100	/	/	/	NA	/	/	/	83	/	/	/	100	/	/	/	0	/	/	/	100
Minor error	/	/	/	0	/	/	/	0	/	/	/	0	/	/	/	0	/	/	/	2	/	/	/	0
Major error	/	/	/	0	/	/	/	0	/	/	/	1	/	/	/	0	/	/	/	0	/	/	/	0
Very major error	/	/	/	0	/	/	/	0	/	/	/	0	/	/	/	0	/	/	/	0	/	/	/	0

^a^ Agreement between RAST and the reference method (Phoenix). Ceftazidime (CAZ), piperacillin/tazobactam (TZP), meropenem (MEM), imipenem (IMI), ciprofloxacin (CIP), amikacin (AMK), area of technical uncertainty (ATU). NA: not applicable because of all the readings fell to ATU.

**Table 5:** Categorical agreement (%) for each pathogen–antibiotic combination. Ranges reflect variation in CA across incubation times (4, 6, 8, 16–20 h).

Pathogens	AMK	AMP	TZP	CAZ	IMI	MEM	CIP	TMP-SMX
*E. coli*	88–100	75–100	88–100	75–100	100	75–100	88–100	50–75
*K. pneumoniae*	89–100	-	100	100	100	100	89–100	67–90
*A. baumannii*	100	-	-	-	100	100	100	100
*P. aeruginosa*	100	-	0	100	-	83	100	-

**Table 6:** VME and ME absolute frequencies and rates according to specific pathogen and reading time.

Pathogen	Very Major Error Number and Rate (%)				Major Error Number and Rate (%)			
	4 h	6 h	8 h	16/20 h	4 h	6 h	8 h	16/20 h
*E. coli*	2	6	6	1	0	1	1	1
	(8.7%)	(26.1%)	(26.1%)	(4.3%)	(0.0%)	(0.9%)	(0.9%)	(0.9%)
*K. pneumoniae*	0	2	2	1	1	1	1	4
	(0.0%)	(1.8%)	(1.8%)	(0.9%)	(2.4%)	(2.4%)	(2.4%)	(9.7%)
*A. baumannii*	0	1	1	0	0	0	0	0
	(0.0%)	(1.5%)	(1.5%)	(0.0%)	*	*	*	*
*P. aeruginosa*	NA	NA	NA	0	NA	NA	NA	1
				(0.0%)				(8.3%)

* 0 cases of susceptibility according to Phoenix system were detected then ME rate was not calculable (0/0). NA: not applicable, all readings were performed at 16/20 h.

## Data Availability

Data are available on request to corresponding authors.

## Abbreviations

The following abbreviations are used in this manuscript:

AMK	Amikacin
AMP	Ampicillin
AST	Antimicrobial susceptibility test
ATU	Area of technical uncertainty
BSI	Bloodstream infection
CA	Categorical agreement
CAZ	Ceftazidime
CAZ-AVI	ceftazidime–avibactam
CIP	Ciprofloxacin
CRKP	Carbapenem-resistant *K. pneumoniae*
ESKAPEEc	*Enterococcus faecium*, *Staphylococcus aureus*, *Klebsiella pneumoniae*, *Acinetobacter baumannii*, *Pseudomonas aeruginosa*, *Enterobacter* spp., and *Escherichia coli*
EUCAST	European Committee on Antimicrobial Susceptibility Testing
GEN	Gentamicin
IMI	Imipenem
MALDI-TOF MS	Matrix-Assisted Laser Desorption/Ionization–Time-of-Flight Mass Spectrometry
mE	Minor error
ME	Major error
MEM	Meropenem
MIC	Minimum inhibitory concentration
PCR	Polymerase chain reaction
RAST	Rapid antimicrobial susceptibility test
TMP-SMX	Trimethoprim–sulfamethoxazole
TZP	Piperacillin tazobactam
VME	Very major error
